# Experimental study on the axial tensile properties of polypropylene fiber reinforced concrete

**DOI:** 10.1038/s41598-023-43723-5

**Published:** 2023-09-29

**Authors:** Xutao Zhang, Ruijie Yin, Yunjuan Chen, Chao Lou

**Affiliations:** 1 School of Architecture and Engineering, Liaocheng University, Liaocheng, 252059 China; 2 Civil Engineering College, Shandong Jianzhu University, Jinan, 250101 China

**Keywords:** Civil engineering, Mechanical properties, Composites

## Abstract

In order to study the axial tensile properties of polypropylene fiber reinforced concrete, an axial tensile test device for concrete is developed in this paper. The device is composed of three parts: rigid frame, spherical hinge and puller, and specimen fabrication part. The test device can accurately measure the tensile strength and peak tensile strain of concrete, and perfectly solves the eccentricity problem of concrete specimens under tension. It can measure the post peak segment tensile strain, such that the whole process tensile stress–strain curve can be obtained. The axial tensile test of polypropylene fiber concrete was carried out using the above test device, and the results show that the tensile strength of concrete can be clearly improved by adding polypropylene fiber, which makes the tensile failure of concrete show certain plastic characteristics. The test results show that with the increase in fiber content, the tensile strength of concrete increases first and then decreases. The effects of polypropylene fiber content and curing age on the tensile properties of concrete were studied and the optimum polypropylene fiber content was determined. When the fiber content is 0.9 kg/m^3^, the tensile strength of concrete reaches the maximum value. The splitting tensile test of concrete under the same condition was carried out simultaneously. The damage phenomenon and test results of the axial tensile test and splitting tensile test of concrete were compared and analyzed, and the applicability of the new developed device in the concrete axial tensile test was verified.

## Introduction

The addition of polypropylene fibers to concrete can provide significant benefits, improved economy and sustainability. For example, the combination of fiber and concrete can enhance the crack resistance of concrete^1,2^. Polypropylene fiber has excellent mechanical properties, good corrosion resistance and low price. Adding a proper amount of polypropylene fiber into concrete can obviously improve the mechanical properties and crack resistance of concrete^3–5^. The plastic shrinkage of concrete can be reduced by 12–25% when 0.1–0.3% polypropylene fiber is added. In addition, the splitting tensile strength and flexural strength of concrete after 7 and 28 days can be increased, and the tensile–compressive ratio at 28 days can be increased by 46%. Obviously, polypropylene fibers can significantly improve the performance of concrete^6–8^.

At present, most of the researches on the mechanical properties of concrete are focused on the performance of concrete under compression and bending loads, while studies on the axial tensile behavior of concrete have been rare. At present, the tensile strength of concrete is mainly measured by the indirect method, including splitting test^9,10^ and bending test^11^. The tensile strength is calculated according to the empirical formula, whereas the internal stress state of the specimen is more complicated; the error of the measured tensile strength is larger, and the ultimate tensile strain and the tensile stress–strain curve of concrete cannot be measured^12^. The axial tensile test is the most suitable method to test the tensile strength of concrete^13^. Several scholars have performed experiments in this regard.

Julia et al.^14^ conducted a three-point bending test and an axial tensile test at the same time. In the axial tensile test, cylindrical concrete specimens were used. Moreover, an axial tensile testing device was developed. The two ends of the specimens were clamped by a metal fixture to exert tensile force, but this approach does not take any measures to eliminate eccentricity. Kim et al.^15^ used a dumbbell specimen with variable section, and the stress concentration was reduced by the variable section, so that the middle part of the specimen was a uniform tensile zone. Liao et al.^16^ designed a set of concrete axial tensile device and clamp and carried out direct and indirect tensile tests on concrete at the same time. Kasagani and Rao^17^ developed an external clamp-type concrete fixture that secures the upper and lower ends of concrete and sets a pull ring at both ends to eliminate the eccentricity of the specimen. This method was used to measure the rising section of tensile stress–strain curve of concrete. The experimental work was carried out under uni-axial tension with 0.1%, 0.2%, 0.3%, 0.4%, 0.50% fiber volumes of Mono Glass Fibers, and the results showed that the strength, deformation capacity and energy absorption capacity were higher for Graded Glass Fiber Reinforced concrete than for Mono Glass Fiber Reinforced Concrete.

Chrysanidis and Panoskaltsis^18^ designed prismatic specimens with embedded steel bars, in which the steel bars on both ends were welded to steel plates to exert tension on the concrete. Chun and Yoo^19^ used dog-bone concrete specimens for the tensile strength tests of steel fiber reinforced concrete. The size of specimens was small, and the width of tension zone was only 25 mm. It was found that the tensile test is easily affected by eccentricity and size effects.

In summary, the main problems of concrete axial tensile test device are as follows: (1) the size of the specimen is too small and the shape of the specimen is unreasonable, and the test results are affected by the size effect and stress concentration. The internal tensile stress of the test piece is not uniform, which leads to the distortion of test results. (2) Eccentricity will easily occur during the test. (3) The concrete will break immediately after cracking, thus the descending segment of the tensile stress–strain curve cannot be measured.

In order to solve the current challenges in the axial tensile test of concrete, a set of concrete axial tensile test equipment was developed in this work. The axial tensile test of polypropylene fiber concrete was carried out using this device, and the tensile strength, peak tensile strain and the whole process curve of tensile stress–strain were determined. Based on the test data, the effect of polypropylene fiber on the tensile properties of concrete was analyzed. The axial tensile test and splitting tensile test of concrete were carried out simultaneously under the same condition. Finally, according to the stress state inside the specimen and the test data, the applicability of the new test device in the study of the tensile properties of concrete was verified.

## Construction of axial tensile test device for concrete

Three common test methods exist for the concrete axial tension test. The first test method uses some kind of fixture. The clamp is fixed outside the concrete specimen, and the tension is transmitted by the friction force with the concrete surface^20^. The second test method involves embedding a reinforcement at both ends of the concrete specimen. The uniform tensile zone is formed in the middle of the specimen, and the tensile force is transmitted through the adhesive force between the steel bar and concrete^21,22^. In the third test method, also called the bonding method, the steel plate is pasted on both ends of the concrete specimen, and the tensile force is directly applied to the specimen^23,24^. However, all of these three methods indirectly test the tensile strength and have obvious defects. Thus, the failure of the test will easily occur, and the downward section of the stress–strain curve cannot be obtained.

**Figure 1:**
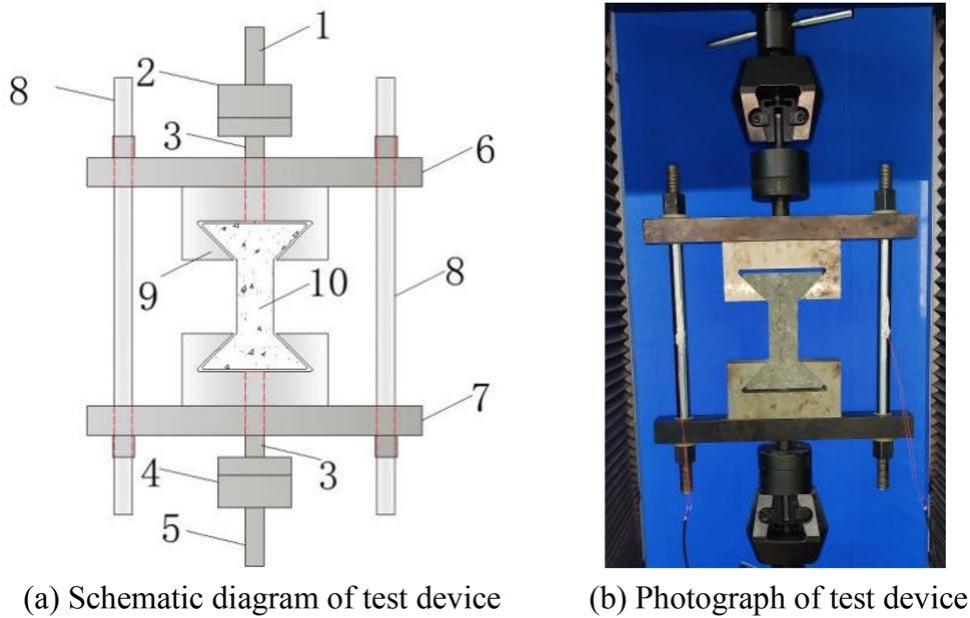
Axial tensile test device for concrete. (In the figure: 1—upper pull rod; 2—upper spherical hinge; 3—connecting rod of spherical hinge; 4—lower spherical hinge; 5—lower pull rod; 6—upper beam; 7—lower beam; 8—round steel; 9—puller; 10—concrete specimen).

The success of concrete axial tensile test mainly depends on two aspects: the first is whether the eccentricity of the specimen can be avoided, and the second is whether the stress concentration of the specimen can be solved^25^. Herein, based on the above analysis, a set of axial tension test device is developed. The device is composed of three parts: rigid frame, spherical hinge and puller, and specimen fabrication part, as shown in Fig. 1.

### Rigid frame part

**Figure 2:**
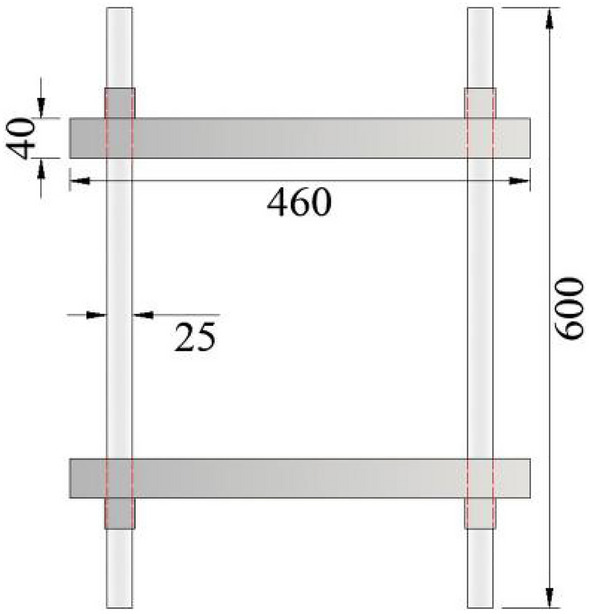
Schematic diagram of rigid frame part.

Figure 2 illustrates the schematic diagram of the rigid frame. This part mainly includes two beams and two round bars, and the round steel is connected and fixed with the beam through nuts at both ends. The length of the beam is 460 mm, the width is 50 mm, and the thickness is 40 mm. The diameter of round steel is 25 mm and the length is 600 mm. During the test, the round steel and the concrete specimen are pulled together, which solves the difficulty of measuring the tensile stress–strain curve after the peak strength of concrete.

### Spherical hinge part and puller

There are two main reasons for eccentricity in the axial tensile test: one is the deviation of the specimen placement position, and the other is the error of the test device itself because the pullers in the test device are not on the same axis.

**Figure 3:**
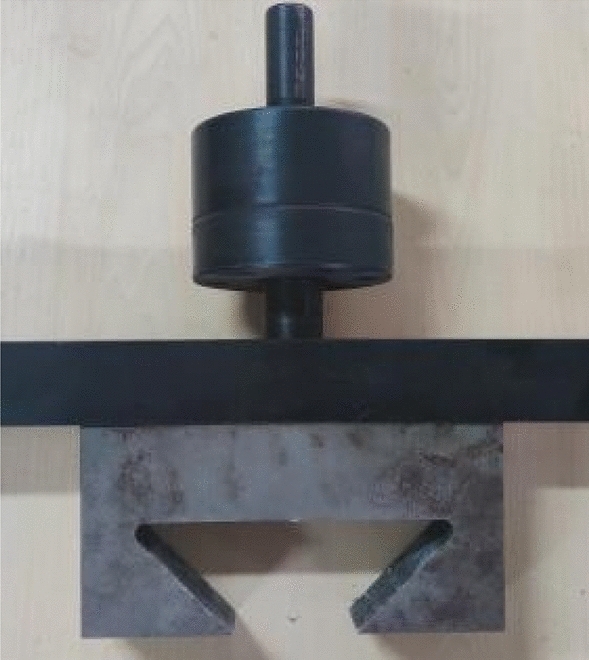
Spherical hinge part and puller.

In order to solve the eccentricity problem, the spherical hinge part and puller are designed, as shown in Fig. 3. The spherical joint is located between the concrete specimen and the puller of the testing machine, and can rotate freely within a certain range. During the test, it can be ensured that the tensile force and the axis of the concrete specimen are always on the same axis, which eliminates the eccentricity problem.

### Specimen fabrication

**Figure 4:**
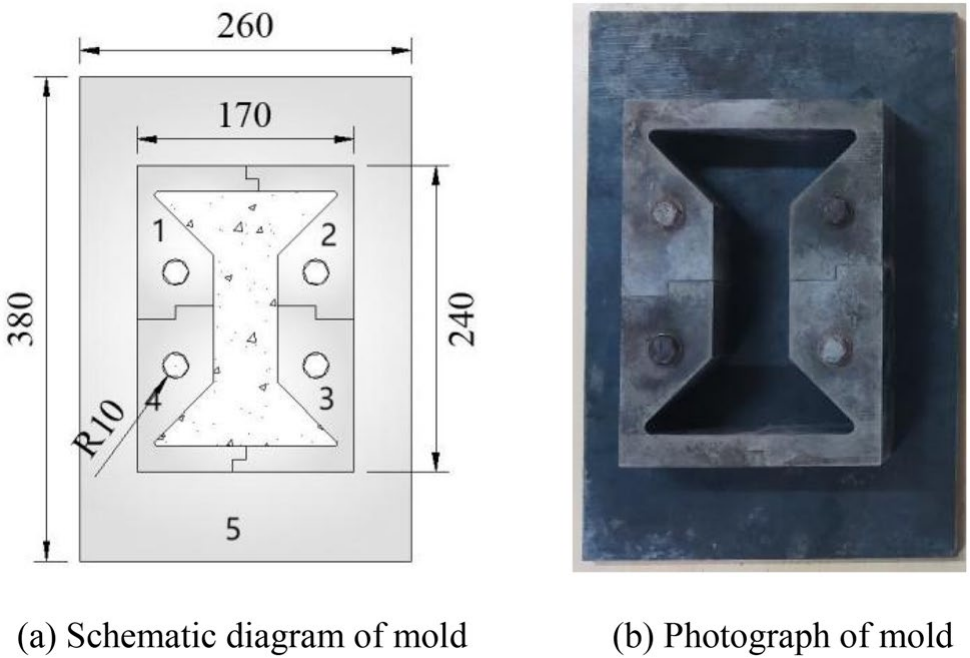
Concrete specimen mold.

Considering that concrete is easy to be poured and formed, a dumbbell-shaped concrete specimen and mold are designed. The dimensions of the mold and concrete specimen are shown in Fig. 4. The central part of the concrete specimen is the tensile zone, the length of the tensile zone is 100 mm, the width is 50 mm, and the thickness is 50 mm. The mold is composed of four side plates and one bottom plate, wherein the side plates are fixed on the bottom plate by bolts. When making concrete specimens, the mold is assembled first, and then the concrete is poured in it. After the initial setting of concrete, the mold is disassembled and the specimen is allowed to cure. The dumbbell-shaped concrete specimen is perfectly matched with the puller, which is convenient for applying tension to the specimen and eliminating stress concentration, and effectively improves the accuracy and success rate of the test. In addition, a 3-mm gap is reserved between the puller and specimen for easy installation of the specimen. A rubber pad is set at the position where the puller contacts the specimen to eliminate the stress concentration caused by local contact.

### Advantages of the proposed concrete axial tensile test device

Compared with other tensile test devices, this test device provides the following benefits: (1) the device is easy to install and disassemble. When used with the universal testing machine, the tensile strength, peak tensile strain and the whole-process tensile stress–strain curve of concrete can be accurately measured. (2) The operation of tensile test is simple, and the mold can be designed as needed for making a concrete specimen. The preparation of the specimen can be done easily. The dumbbell-shaped specimen is convenient for transferring the tensile force and forming a uniform tensile zone in the middle of the specimen. The measured tensile strength is closer to the true value. (3) The rigid frame in the device and the specimen are pulled at the same time, and the post-peak section tensile stress–strain curve of concrete can be measured. (4) The spherical joint in the device effectively eliminates eccentricity, which makes the specimen always keep the axial tension state. (5) The device is not only limited to testing the tensile strength of concrete, but also can be used to assess the tensile strength of other easily formed materials; hence, it has potential for wide application.

## Application of the proposed concrete axial tensile test device

In order to directly test the tensile strength, peak stress and full tension stress–strain curve of polypropylene fiber reinforced concrete, the axial tensile test is carried out using the newly developed test device.

### Fabrication of concrete specimen

The design strength grade of polypropylene fiber reinforced concrete is C30, the cement is P.O 42.5 ordinary portland cement, the particle size of gravel is 5–20 mm, the fineness modulus of fine aggregate is 2.5, and the coarse and fine aggregates are well graded. The concrete mix proportion is as follows, cement:water:sand:gravel = 337.95:4.875:634.79:1232.25. The mechanical properties of polypropylene fiber are summarized in Table 1.

**Table 1:** Mechanical properties of polypropylene fiber.

Type of fiber	Tensile strength/MPa	Modulus of elasticity/GPa	Elongation at break/%	Diameter/μm	Density/(g/cm^3^)
Polypropylene fiber	556.9	4.09	26.8	31	0.91

The amounts of cement, water, sand and fiber are calculated and determined. Before the pouring of the sample, the coarse aggregate was first cleaned and air-dried. In the mixing process, the coarse aggregate and sand were added at first, the mixture was stirred for 60 s, then cement and half water were added, the mixture was stirred for 30 s, then polypropylene fiber was added twice, and the mixture was stirred for 60 s. Finally, the other half water and water-reducing agent were added to stir the mixture for 180 s, and water and polypropylene fiber were added in two stages to achieve the required stirring state. Then the slump of each sample was measured immediately after mixing. Subsequently, the mixed concrete is poured into dumbbell-shaped and cube-shaped molds, which are vibrated evenly on a vibrating table. After the completion of the casting and demoulding, the sample should be immediately moved to the standard concrete curing room where the temperature and humidity are constant, the temperature of the curing room is constant at 20 ± 2 °C, and the relative humidity is not less than 95%. During curing, the specimens are protected from damage.

### Test variables

In the axial tensile test of polypropylene fiber concrete, two factors are mainly considered: the content of polypropylene fiber and curing age. The length of the polypropylene fiber is 19 mm. Six different fiber contents are designed, namely, 0 kg/m^3^, 0.3 kg/m^3^, 0.6 kg/m^3^, 0.9 kg/m^3^, 1.2 kg/m^3^, and 1.5 kg/m^3^. Three different curing ages are considered, namely, 14 days, 28 days and 60 days. Based on the above two factors, 18 groups of specimens are prepared. Each group of specimens includes 4 dumbbell-shaped specimens and 6 cube specimens. The dumbbell-shaped specimen is fabricated using the above mold, and the side length of the cube specimen is 150 mm.

### Steps of axial tensile test

**Figure 5:**
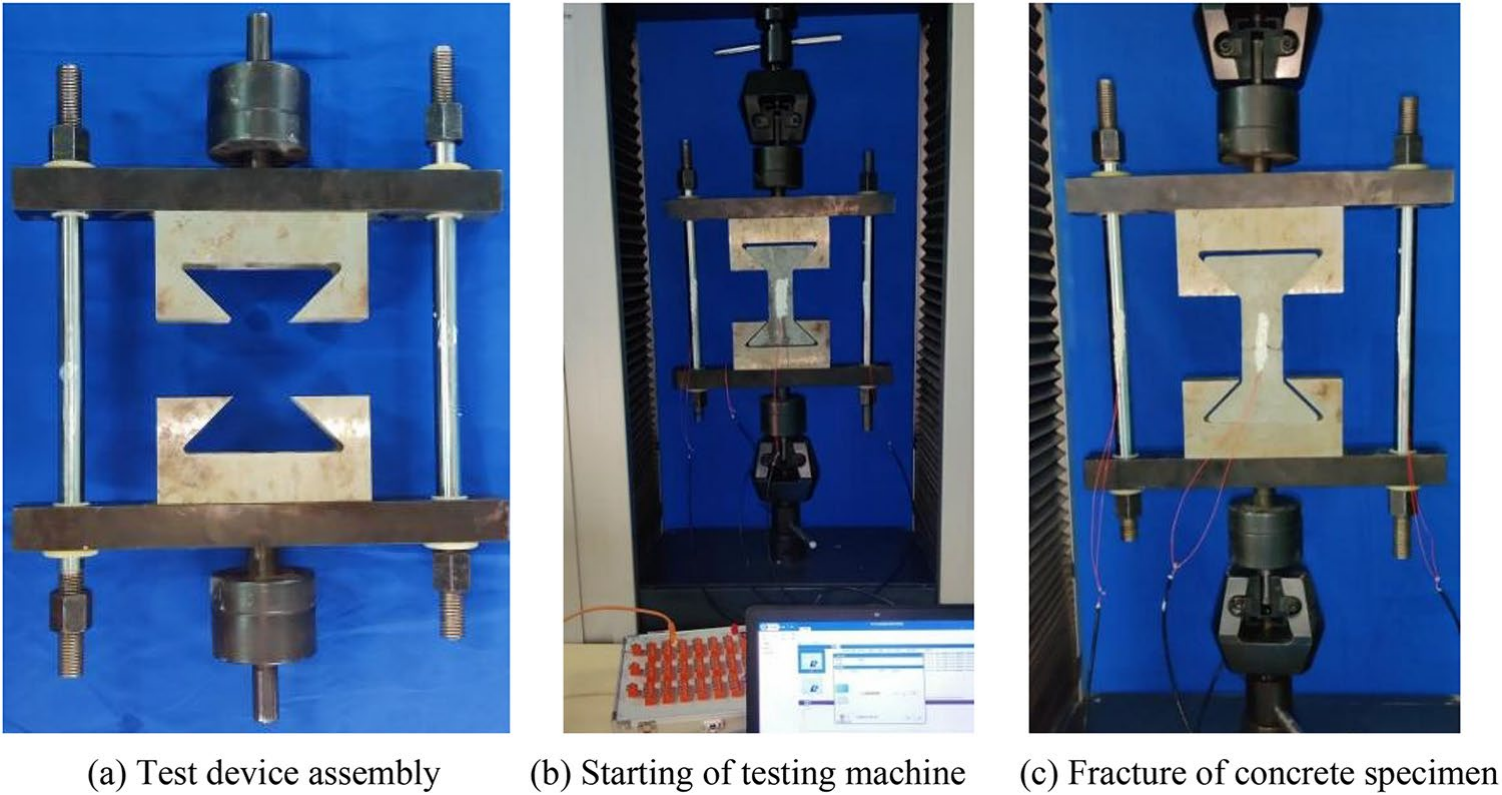
Steps of axial tensile test.

The axial tensile test of concrete is carried out using the above device. The main test steps are shown in Fig. 5. (1) The test device is assembled. According to the drawing of assembly (Fig. 1), the rigid frame part, spherical joint part and upper and lower puller of the device are assembled together. (2) The testing device is adjusted, as well as the tensile space of the universal testing machine. The upper and lower pull rods of the device are respectively connected with the clamps of the testing machine and then clamped. The strain gauge is pasted on the round steel on both sides and tested with electricity. (3) Concrete specimens are installed. The specimen is taken out from the curing box and tested after its surface is dry. It is then installed between the upper and lower puller of the axial tension device. The strain gauges are pasted on the front and rear surfaces of the specimen. The testing machine is started, a small tensile force is applied first, and the strain of round steel on both sides is observed, ensuring that it has the same value on both sides. If this is not the case, the fastening bolts on the round steel are adjusted, and the tensile force is reduced to zero after commissioning. (4) The specimen is loaded. The testing machine is started, tension is applied in the form of equal strain, and loading is continued until the test piece is completely broken. The tensile force *F* of the testing machine, the tensile strain *ε*_0_ of the round steel, and the tensile strain *ε* of the concrete specimen are recorded. The tensile force *F*_0_ of the concrete specimen is:

1 $${F}_{0}=F-{E}_{0}{\varepsilon }_{0}A$$

In the above formula, *E*_0_ denotes the elastic modulus of the round steel, *A* denotes the cross-sectional area of the round steel on both sides, and *ε*_0_ is the tensile strain of the round steel.

The axial tensile strength of concrete specimens is:

2 $${f}_{t}=\frac{{F}_{0}}{{A}_{c}}$$

In the above formula, *A*_*c*_ denotes the cross sectional area in the middle of the concrete specimen.

Based on the above test data, the tensile strength, peak tensile strain and full process tensile stress–strain of polypropylene fiber concrete are obtained.

## Axial tensile test results and analysis of polypropylene fiber reinforced concrete

### Axial tensile test results

Eighteen groups of axial tensile tests of fiber reinforced concrete were carried out using our self-developed axial tensile test device. The test results are shown in Table 2. The numbering rules of the specimens are as follows: PFRC stands for polypropylene fiber reinforced concrete, and the first number 14, 28 and 60 after PFRC represent the curing ages of specimens as 14 days, 28 days and 60 days, respectively. The second numbers 0, 0.3, 0.6, 0.9, 1.2 and 1.5 represent the polypropylene fiber contents of 0 kg/m^3^, 0.3 kg/m^3^, 0.6 kg/m^3^, 0.9 kg/m^3^, 1.2 kg/m^3^, and 1.5 kg/m^3^, respectively.

**Table 2:** Tensile strength of polypropylene fiber reinforced concrete.

Specimen number	Axial tensile strength *f*_t_/MPa	Peak tensile strain *ε*/10^–6^	Splitting tensile strength *f*_ts_/MPa
PFRC-14-0	1.33	118.44	2.00
PFRC-14-0.3	1.55	113.52	2.02
PFRC-14-0.6	1.69	120.96	2.04
PFRC-14-0.9	1.97	139.23	2.18
PFRC-14-1.2	1.91	148.5	2.09
PFRC-14-1.5	1.78	130.62	1.95
PFRC-28-0	1.47	191.10	2.10
PFRC-28-0.3	1.72	224.10	2.17
PFRC-28-0.6	1.87	272.40	2.32
PFRC-28-0.9	2.11	264.90	2.52
PFRC-28-1.2	2.04	286.56	2.45
PFRC-28-1.5	1.90	295.50	2.38
PFRC-60-0	1.58	190.35	2.17
PFRC-60-0.3	1.76	155.25	2.27
PFRC-60-0.6	1.92	257.85	2.33
PFRC-60-0.9	2.20	261.00	2.86
PFRC-60-1.2	2.21	258.30	2.81
PFRC-60-1.5	2.05	314.64	2.64

### Influence of polypropylene fiber content and curing age on the tensile strength of concrete

**Figure 6:**
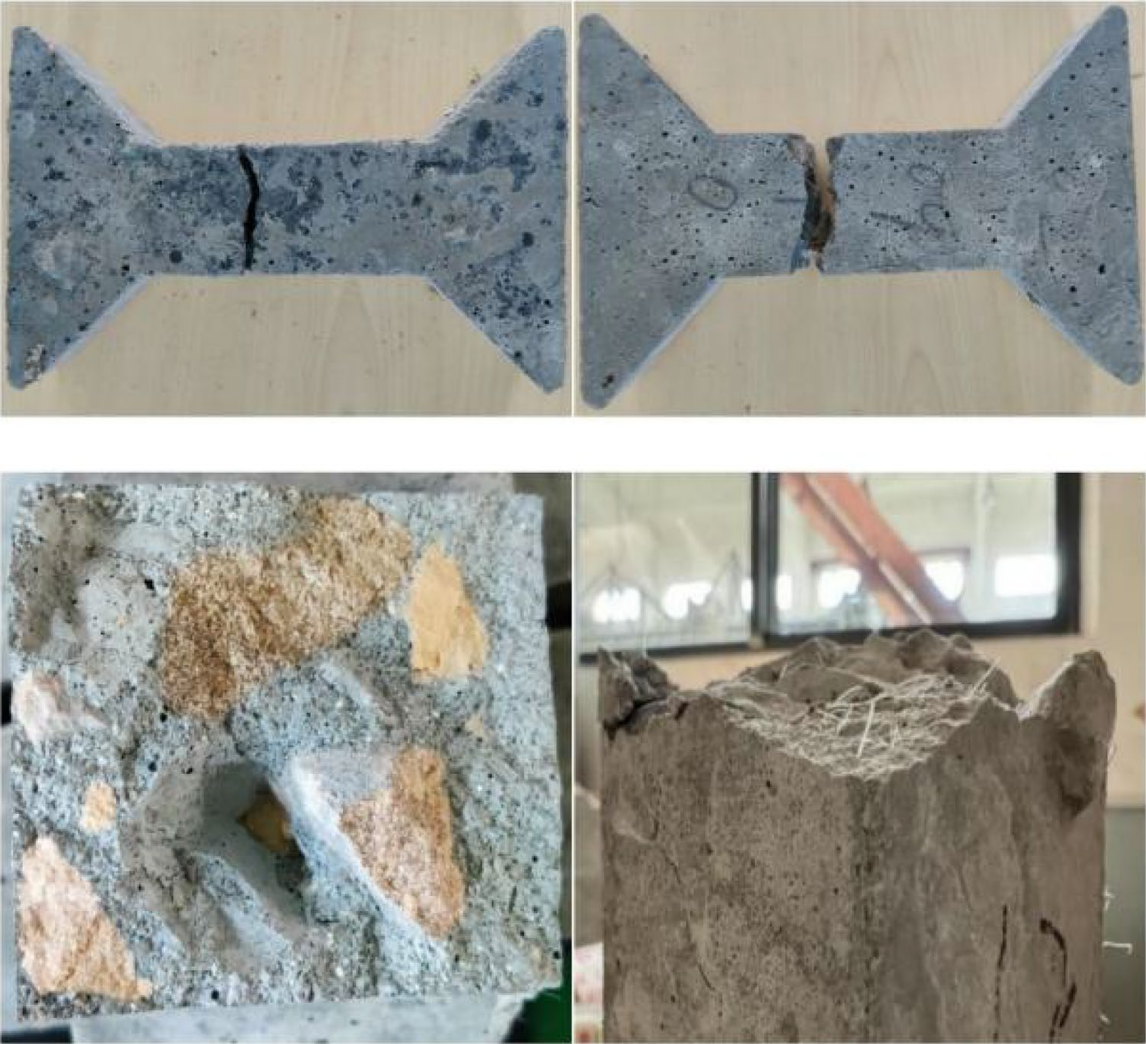
Failure mode of polypropylene fiber concrete.

**Figure 7:**
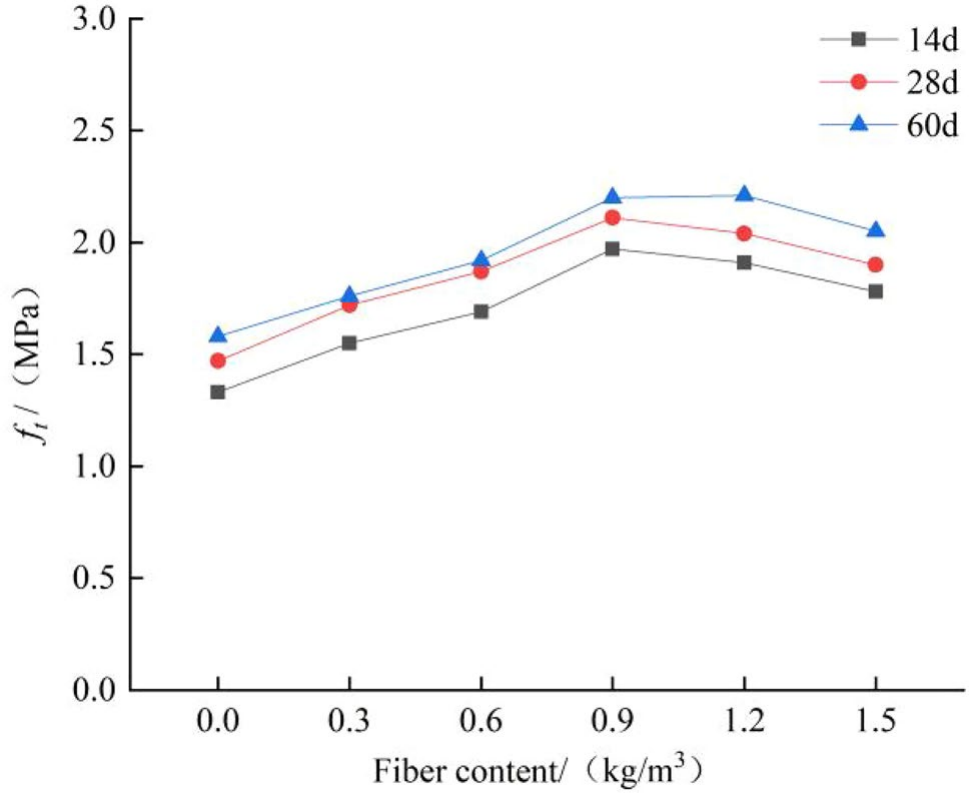
Effect of polypropylene fiber content on tensile strength.

As shown in Fig. 6, the fracture position of most of the specimens is in the central tensile zone. The fracture surface is flat and basically perpendicular to the axis of the specimens. There is no protruding coarse aggregate on the fracture surface. When the fiber content is above 0.9 kg/m^3^, the pulled polypropylene fiber can be observed on the fracture surface. The change law of concrete tensile strength with the polypropylene fiber content is shown in Fig. 7. Based on the tensile strength of plain concrete, when the fiber content is 0.3 kg/m^3^, 0.6 kg/m^3^, 0.9 kg/m^3^, 1.2 kg/m^3^ and 1.5 kg/m^3^, the tensile strength is increased by 17%, 27%, 43%, 38%, and 29%, respectively. With the increase in fiber content, the tensile strength of concrete rises first and then declines. When the fiber content is 0.9 kg/m^3^, the increase in the tensile strength of concrete is the most obvious.

The uniformly distributed polypropylene fiber will connect the weak area between the cement matrix and the aggregate. When the concrete is in tension, the fiber will share part of the tension, which improves the tensile strength of the concrete. Because the fiber is non-directionally distributed in the concrete, when the fiber content is large, the fiber agglomeration phenomenon will occur in the concrete^26^, and bubbles and irregular holes will appear at the fiber overlap, which in turn increases the probability of internal defects in the concrete, thus the tensile strength of the concrete is reduced.

**Figure 8:**
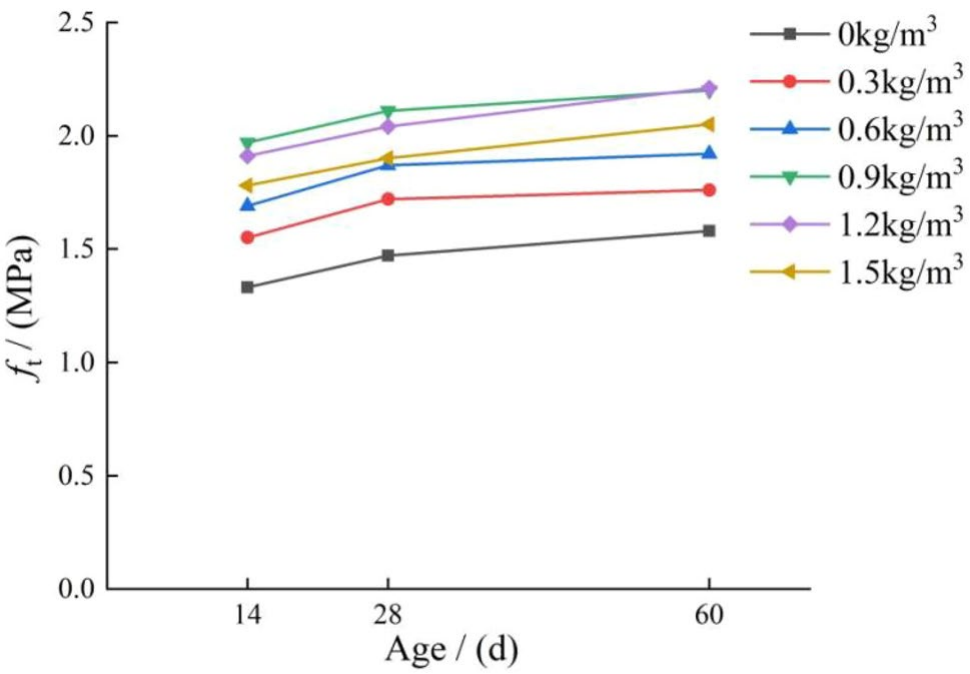
Effect of curing age on the tensile strength of concrete.

The influence of curing age on the tensile strength of concrete is shown in Fig. 8. It can be seen from the figure that the tensile strength of polypropylene fiber concrete monotonously increases with the curing age, then the growth rate slows down to a certain extent and gradually converges. There are two specific reasons for this phenomenon: firstly, the internal hydration reaction of concrete specimens after forming is intense, and the early strength development speed is faster. In the later stage of specimen curing, the hydration reaction becomes gradually weaker, and the growth rate of concrete strength slows down until it stabilizes. Secondly, the addition of polypropylene fibers improves the internal structure of concrete, resulting in more complete hydration reactions and the rapid growth of early strength.

The strength of polypropylene fiber concrete increases rapidly in the early stage. At the curing time of 14 days, the tensile strength has reached more than 90% of the tensile strength of that at 28 days. When the fiber content is 0 kg/m^3^, 0.3 kg/m^3^, 0.6 kg/m^3^, 0.9 kg/m^3^, 1.2 kg/m^3^, and 1.5 kg/m^3^, the ratio of 14 days tensile strength to 28 days tensile strength is 0.904, 0.901, 0.903, 0.933, 0.936, and 0.936 respectively. The tensile strength at 60 days is increased very little; compared with the tensile strength at 28 days, it is increased by 7.4%, 2.3%, 2.6%, 4.2%, 8.3%, and 7.8%, respectively. The tensile strength of concrete with different fiber contents remains stable with the curing age. Therefore, it can be considered that the tensile strength of polypropylene fiber concrete increases rapidly in the first 14 days. During this period, the hydration reaction inside the concrete is intense, which is the key period for the performance improvement of concrete. After 14 days, the tensile strength of polypropylene fiber concrete does not increase much and basically reaches stability.

### Effect of polypropylene fiber on the tensile stress strain curve of concrete

**Figure 9:**
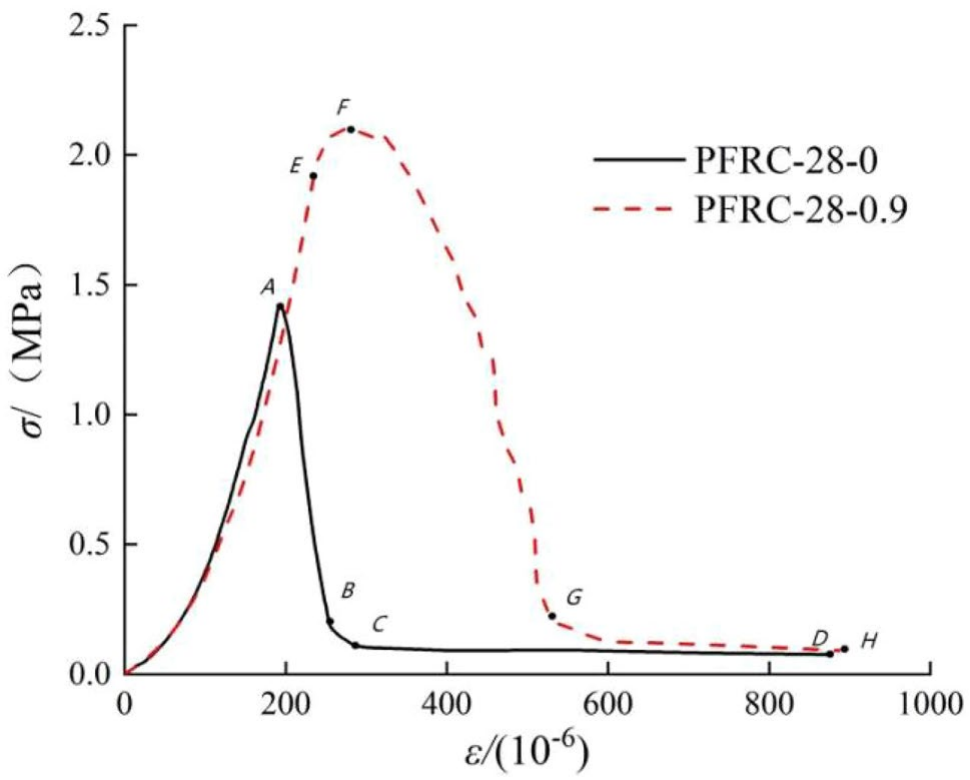
Tensile process comparison between fiber reinforced concrete and ordinary concrete.

The typical stress–strain curves of polypropylene fiber reinforced concrete and ordinary concrete are shown in Fig. 9. The numbers of the two curves are PFRC-28-0.9 and PFRC-28-0, corresponding to the concrete with a polypropylene fiber content of 0.9 kg/m^3^ and without polypropylene fiber, respectively. The reason for choosing PFRC-28-0.9 is that when the curing age is 28 days, the mechanical properties of concrete are improved the most when the content of polypropylene fiber is 0.9 kg/m^3^, and the stress–strain curve of the specimen is the smoothest and most representative.

The tensile stress–strain curve of concrete can be divided into the following stages: elastic stress stage, crack propagation stage, falling stage, and failure stage, so as to show the whole tensile process of fiber reinforced concrete.

Elastic stress stage: this corresponds to the OA and OE sections, where the stress–strain curve is close to a straight line, and the stress growth rate is fast. In the OE stage, the polypropylene fiber and concrete matrix are under tension together, so the polypropylene fiber concrete can bear greater tensile force than ordinary concrete.

Fracture propagation stage: this corresponds to the *EF* segment of the curve and is the microcrack development stage. The stress–strain curve appears an inflection point at E, and the slope decreases. At this time, microcracks in the concrete gradually expand and the strain increases. With the continuous increase in tensile stress, microcracks develop to a certain extent, and the polypropylene fiber begins to bear tensile force, which prevents the further development of microcracks. The microcracks in the specimen gradually connect and penetrate to form the main crack, the force transmission path in the concrete decreases, and the stress of the specimen declines. At this time, the polypropylene fibers mainly play the role of crack resistance. In this stage, the stress–strain curve begins to become gentle and the growth rate of strain is accelerated. Compared with polypropylene fiber reinforced concrete, ordinary concrete has no obvious crack propagation stage, and when the tensile stress reaches the peak stress value, the fracture surface quickly forms and the tensile capacity is lost, which also leads to the peak strain of ordinary concrete being lower than that of polypropylene fiber reinforced concrete. Some concrete specimens with low fiber content will also break quickly after reaching the peak stress and show no obvious falling curve. This is because of the low fiber content. In addition, the tensile strength of the fiber and the bonding force with the concrete are insufficient, so the improvement of the tensile performance of the concrete is limited.

Descent phase: this corresponds to the *FG* and *AB* stages of the stress–strain curve. At this stage, the stress–strain curve of polypropylene fiber concrete will drop in a fluctuating manner, because the fiber at the fracture surface will be pulled off or pulled out after being subjected to the tensile force. However, the falling stage of ordinary concrete is approximately linear without the crack resistance effect of polypropylene fiber, and the specimen is rapidly destroyed.

Failure stage: this corresponds to the GH and CD stages of stress–strain curve. For polypropylene fiber concrete, point G represents the residual stress of fiber concrete. After point G, the stress–strain curve enters the convergence stage, the stress decreases to the minimum and does not change, and the strain increases rapidly. At this time, the specimen is completely destroyed, and most of the fibers are pulled off or pulled out. Ordinary concrete is completely pulled off after point C, and the stress becomes zero.

**Figure 10:**
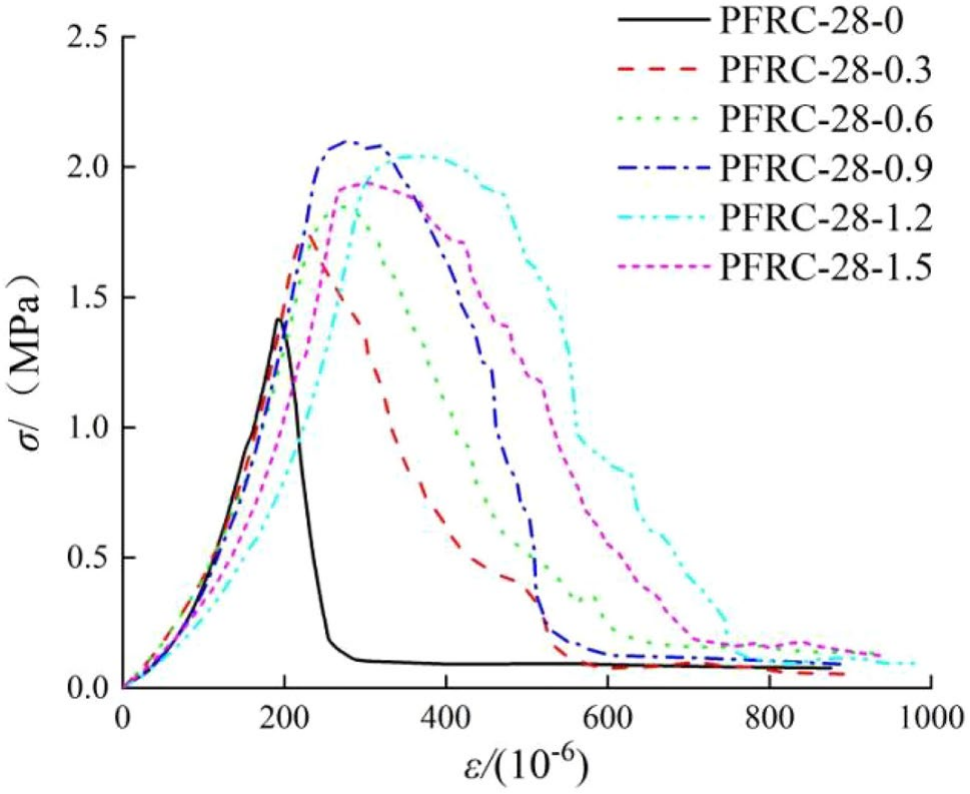
Influence of fiber content on the tensile stress–strain curve.

Figure 10 presents the tensile stress–strain curve of 28 days polypropylene fiber concrete under different fiber contents. The characteristics of the stress–strain curve of ordinary concrete are as follows: at the beginning of loading, the process of the development stage of microcracks is longer because there is no fiber constraint inside, and the development degree of microcracks is higher. Following the end of the microcrack development stage, the slope of the curve becomes larger and the stress rises faster. After reaching the peak value, there is no slow growth process, the crack develops rapidly, and the stress–strain curve shows a sharp point, then decreases linearly in the descending stage.

When the polypropylene fiber content is 0.3 kg/m^3^ and 0.6 kg/m^3^, compared with ordinary concrete, the influence of the fiber content on the tensile stress–strain curve shape is mainly reflected in the following aspects: the development process of microcracks in the elastic stage is obviously shorter, the slope of the curve becomes smaller, and the peak strain increases. The ductility of the concrete is improved after the fiber dimension is added.

When the polypropylene fiber content is 0.9 kg/m^3^, 1.2 kg/m^3^ and 1.5 kg/m^3^, the stress–strain curve shape is more gentle, the curvature of the peak point becomes larger, and the sharp point disappears. There is a relatively obvious slow growth and decline before and after reaching the peak stress. In the descending stage, the higher the fiber content, the more gentle the descending section of the stress–strain curve, the fuller the overall shape of the curve, and the more the residual strength and strain are improved. The residual strength increases slightly with the increase in fiber content, which increase is within 0.2 MPa. When the fiber content is above 0.9 kg/m^3^, the process of stress–strain curve from peak stress to final convergence is longer.

## Comparison and analysis of axial tensile test and splitting tensile test of fiber reinforced concrete

The significance of comparing the axial tension test with the splitting tension test is to point out the difference between an indirect tension test and a direct tension test. It shows the limitation of indirect test represented by the splitting tensile test, whereas the new test device and test method are more reliable and can reflect the true tension state of concrete.

### Splitting tensile test of fiber reinforced concrete

At the time of making dumbbell-shaped concrete specimens, cube specimens were also made with the same mix ratio and fiber content. The side length of the cube specimen is 150 mm, and this specimen is used to carry out the splitting tensile test.

**Figure 11:**
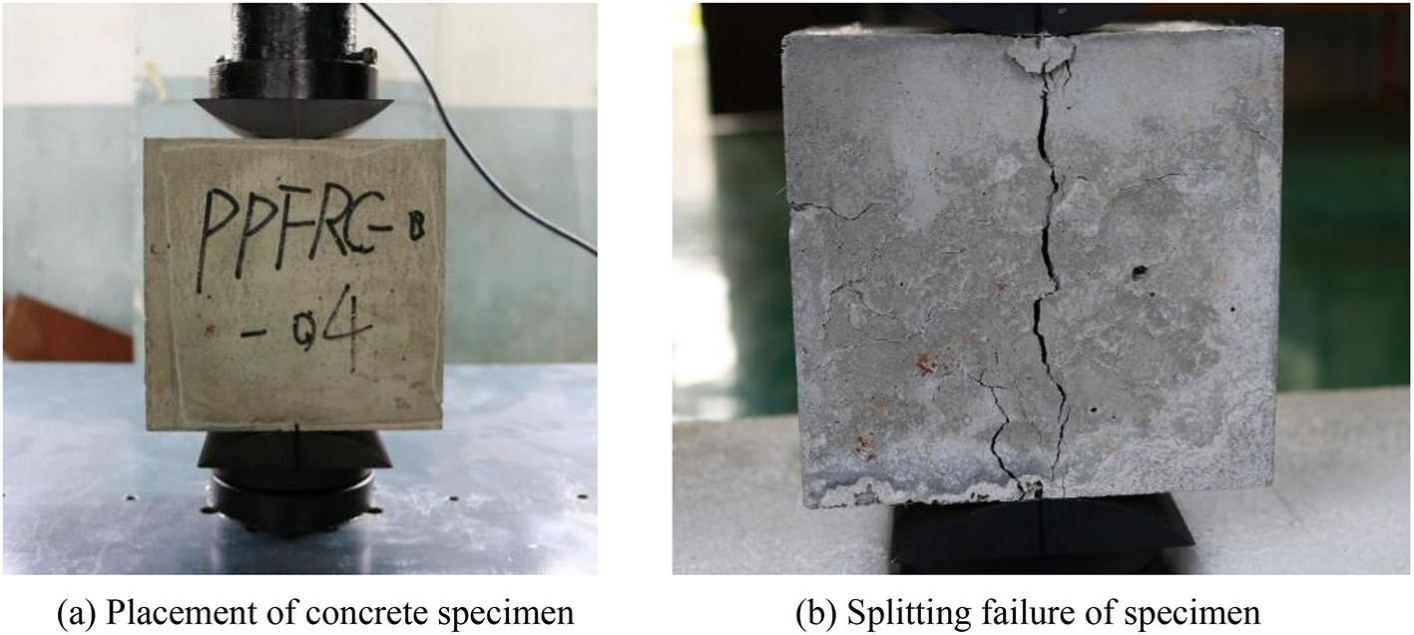
Steps for the splitting tensile test of concrete.

The concrete cube specimen is taken out from the curing box, a line is drawn at the center line of the top and bottom surfaces of the specimen, and the position of the splitting surface is preliminarily determined. The arc cushion block is installed on the universal testing machine. To avoid the influence of eccentricity on the test results, the arc-shaped cushion block is aligned with the centerline of the top and bottom surfaces of the specimen. The specimen is loaded in an equal strain manner until it is destroyed. The pressure F of the testing machine is recorded when the test piece is split. The main steps of splitting tensile test are shown in Fig. 11. The splitting tensile strength of concrete can be calculated by the following formula:

3 $${f}_{ts}=\frac{2F}{\pi A}$$

In the above formula, *f*_*ts*_ denotes the splitting tensile strength of concrete; *F* denotes the pressure when the specimen is split; A is the split surface area of the test piece. The splitting tensile strength of fiber reinforced concrete is shown in Table 2.

### Comparative analysis of axial tensile test and splitting tensile test

**Figure 12:**
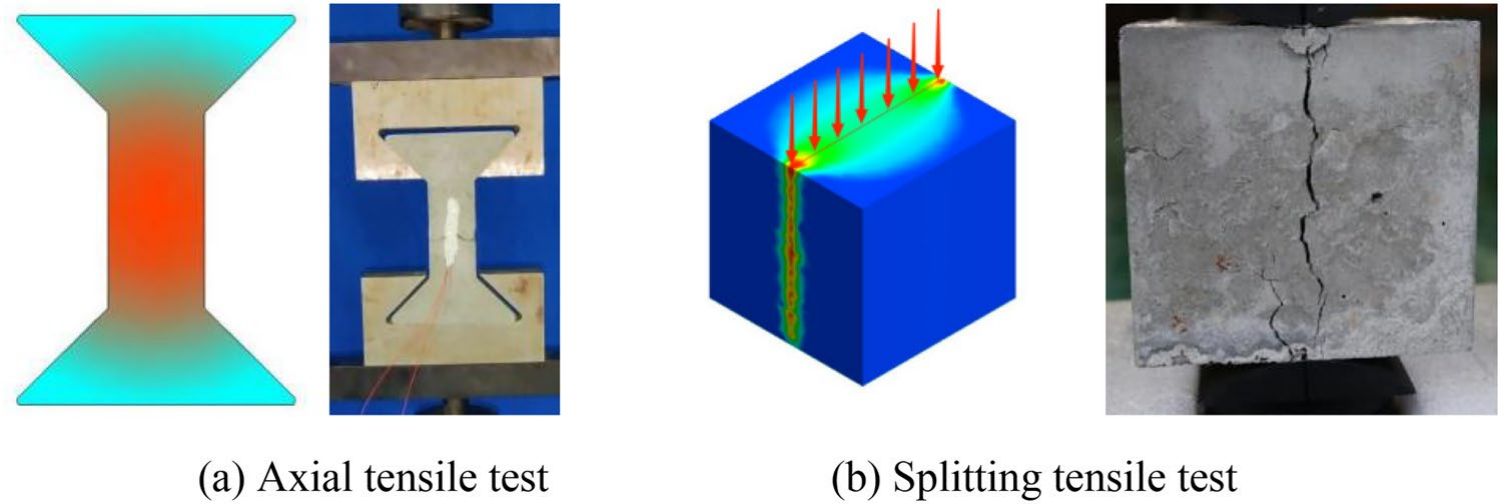
Comparison of axial tensile test and splitting tensile test of concrete.

At present, the splitting tensile test is frequently used to test the tensile strength. The operation is simple and the deviation of test results is small. As shown in Fig. 12, the splitting tensile test is essentially different from the axial tensile test. In the axial tensile test, the tensile zone of the specimen is uniformly stressed, which is a true one-way tensile state. However, in the splitting tensile test, the stress state on the splitting surface of the specimen is more complex; especially, the stress concentration in the area in contact with the arc cushion block is more serious. The splitting tensile strength is derived from the theory of elasticity^27^, which is only an approximate expression of the tensile strength of materials.

**Figure 13:**
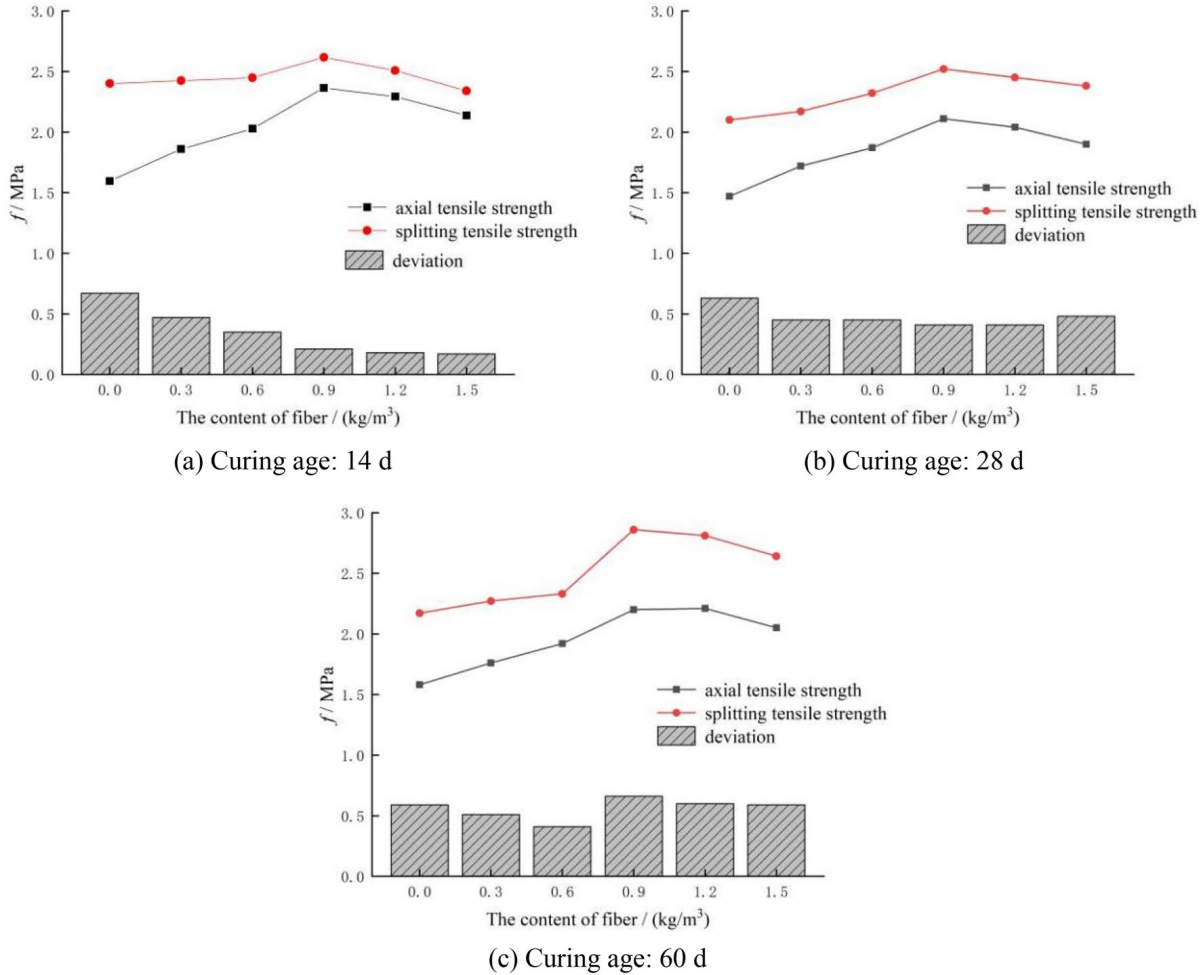
Comparison of axial tensile strength and splitting tensile strength.

When comparing the axial tensile strength and splitting tensile strength of fiber reinforced concrete, it can be seen that there is a deviation between the two tensile strengths; the splitting tensile strength is greater than the axial tensile strength under the same condition. When the curing age is 14 days, the difference between the two tensile strengths is scattered, ranging from 0.17 to 0.67 MPa. Moreover, the difference decreases with the increase in fiber content. When the curing age is 28 days, the difference between the two tensile strengths is relatively average at about 0.5 MPa, and is not affected by the fiber content. When the curing age is 60 days, the difference between the two tensile strengths is relatively large, and the average difference is 0.59 MPa. There is no obvious change law with the fiber content. The change law of the difference between the two tensile strengths and the fiber content is shown in Fig. 13.

The main reasons why the splitting tensile strength is generally higher than the axial tensile strength are as follows:

1. The formula of splitting tensile strength is derived on the assumption that concrete is a homogeneous ideal elastic body. However, the size of sand and gravel aggregate in the concrete is randomly distributed. Concrete is a heterogeneous body formed by cement stone and aggregate bonding, and is not an ideal elastic body. This leads to a deviation between the theoretical results and the real strength.
2. In the splitting tensile test, the failure surface is relatively fixed. It is always on the center line between the upper and lower cushion strips, but this section is not necessarily the weak surface of the concrete. However, the failure surface of the axial tensile test is uncertain, and the crack will appear on a weak surface of the tensile zone in the middle of the specimen. This is the main reason why the axial tensile strength is less than the splitting tensile strength.
3. The production and curing of concrete specimens, the distribution and density of aggregates, the quality of raw materials, the concrete mix ratio, and other test conditions cannot be completely the same. The above factors cause the actual test and theoretical derivation results to be inconsistent.

To sum up, the strength measured by the axial tensile test is the tensile strength of concrete under uniaxial tension, which is the most direct and true result without any theoretical assumptions. Furthermore, it allows the measurement of tensile stress–strain curve of concrete, which cannot be achieved by the splitting tensile test.

## Conclusion

In this paper, to solve the existing problems in the axial tensile test of concrete, an axial tensile test device for concrete is developed. It is composed of three parts: rigid frame, spherical hinge and puller, and specimen fabrication part. The test device is easy to install and disassemble, and can effectively eliminate eccentricity in the test process and avoid stress concentration failure. Combined with a universal testing machine, the tensile strength, peak tensile strain and tensile stress–strain curve of concrete can be accurately measured.

The axial tensile test of polypropylene fiber concrete was carried out by using the newly developed axial tensile test device. The test results show that with the increase in fiber content, the tensile strength of concrete increases first and then decreases. When the fiber content is 0.9 kg/m^3^, the tensile strength of concrete reaches the maximum value. The longer the curing age, the higher the tensile strength of polypropylene fiber concrete, while the tensile strength of polypropylene fiber concrete does not increase after the curing age exceeds 28 days.

The splitting tensile test of concrete under the same conditions was carried out simultaneously. Compared with the axial tensile strength, the splitting tensile strength of concrete is generally larger, the dispersion is high, and there is no obvious regularity. The tensile strain and tensile stress–strain curves of concrete cannot be measured by the splitting tensile test. The applicability of the new developed device in the tensile test of concrete is further verified.

The application of the newly developed test device is not only limited to the tensile strength test of concrete, but also the testing of tensile strength of other easily molded materials. Therefore, the test device can be widely used.

## Data Availability

The authors confirm that the data supporting the findings of this study are available within the article.
